# Scarcity of Women in Victoria

**Published:** 1859-03

**Authors:** 


SCARCITY OF WOMEN IN VICTORIA.—The supply of wives runs short in Victoria. The last returns of the register general of the colony show, that out of a population of 470,000, there are only about 168,000 women to 302,000 men. And the matter is getting worse in this respect; for adult males are still running to the loadstones at the gold fields. 134,000 males are thus left hopeless bachelors; and yet there is no set of men in the world more able or willing to take and maintain a wife, and bear the consequence of marriage. With such facts before us, it is sad to think of the numbers of young women who are daily driven by want and misery into the depths of vice and degradation!—*Ib.*

